# Investigating the Links between Lower Iron Status in Pregnancy and Respiratory Disease in Offspring Using Murine Models

**DOI:** 10.3390/nu13124461

**Published:** 2021-12-14

**Authors:** Henry M. Gomez, Amber L. Pillar, Alexandra C. Brown, Richard Y. Kim, Md Khadem Ali, Ama-Tawiah Essilfie, Rebecca L. Vanders, David M. Frazer, Gregory J. Anderson, Philip M. Hansbro, Adam M. Collison, Megan E. Jensen, Vanessa E. Murphy, Daniel M. Johnstone, David Reid, Elizabeth A. Milward, Chantal Donovan, Jay C. Horvat

**Affiliations:** 1 School of Biomedical Sciences and Pharmacy, College of Health, Medicine and Wellbeing, and Priority Research Centre for Healthy Lungs, University of Newcastle and Hunter Medical Research Institute, Callaghan, NSW 2308, Australia; henry.gomez@newcastle.edu.au (H.M.G.); Amber.pillar@uon.edu.au (A.L.P.); alexandra.brown@newcastle.edu.au (A.C.B.); richard.kim@uts.edu.au (R.Y.K.); mdali@stanford.edu (M.K.A.); rebecca.vanders@newcastle.edu.au (R.L.V.); philip.hansbro@uts.edu.au (P.M.H.); liz.milward@newcastle.edu.au (E.A.M.); chantal.donovan@uts.edu.au (C.D.); 2 Faculty of Science, School of Life Sciences, University of Technology Sydney, Sydney, NSW 2007, Australia; 3 QIMR Berghofer Medical Research Institute, Herston, QLD 4006, Australia; Ama-Tawiah.Essilfie@qimrberghofer.edu.au (A.-T.E.); David.Frazer@qimrberghofer.edu.au (D.M.F.); Greg.Anderson@qimrberghofer.edu.au (G.J.A.); David.Reid@qimrberghofer.edu.au (D.R.); 4 School of Biomedical Sciences, The University of Queensland, St Lucia, QLD 4067, Australia; 5 School of Chemistry and Molecular Bioscience, The University of Queensland, St Lucia, QLD 4067, Australia; 6 Centre for Inflammation, School of Life Sciences, Faculty of Science, Centenary Institute and University of Technology Sydney, Sydney, NSW 2007, Australia; 7 School of Medicine and Public Health, College of Health, Medicine and Wellbeing, and Priority Research Centre for GrowUpWell, The University of Newcastle and Hunter Medical Research Institute, Callaghan, NSW 2308, Australia; Adam.collison@newcastle.edu.au (A.M.C.); megan.jensen@newcastle.edu.au (M.E.J.); vanessa.murphy@newcastle.edu.au (V.E.M.); 8 School of Medical Sciences, University of Sydney, Camperdown, NSW 2050, Australia; daniel.johnstone@sydney.edu.au

**Keywords:** iron deficiency, pregnancy, respiratory disease, asthma, offspring

## Abstract

Maternal iron deficiency occurs in 40–50% of all pregnancies and is associated with an increased risk of respiratory disease and asthma in children. We used murine models to examine the effects of lower iron status during pregnancy on lung function, inflammation and structure, as well as its contribution to increased severity of asthma in the offspring. A low iron diet during pregnancy impairs lung function, increases airway inflammation, and alters lung structure in the absence and presence of experimental asthma. A low iron diet during pregnancy further increases these major disease features in offspring with experimental asthma. Importantly, a low iron diet increases neutrophilic inflammation, which is indicative of more severe disease, in asthma. Together, our data demonstrate that lower dietary iron and systemic deficiency during pregnancy can lead to physiological, immunological and anatomical changes in the lungs and airways of offspring that predispose to greater susceptibility to respiratory disease. These findings suggest that correcting iron deficiency in pregnancy using iron supplements may play an important role in preventing or reducing the severity of respiratory disease in offspring. They also highlight the utility of experimental models for understanding how iron status in pregnancy affects disease outcomes in offspring and provide a means for testing the efficacy of different iron supplements for preventing disease.

## 1. Introduction

Iron deficiency is present in 50% of all pregnancies [1] and nutrient deficiency may be an important aetiological factor. Other aetiologies of iron deficiency include malabsorption diseases such as celiac disease and atrophic gastritis, and blood loss through gastroesophageal reflux and oesophagitis, gastritis, peptic ulcers, inflammatory bowel diseases and menorrhagia [2,3]. Significantly, clinical evidence associates maternal iron deficiency with increased risk of respiratory disease, including asthma, in children [4,5]. Children born to asthmatic mothers with anaemia during pregnancy are more likely to have recurrent wheeze in the first year of life and experience asthma by six years of age [4].

In humans, maternal iron deficiency and increased risk of respiratory disease in offspring implicates iron as a potentially key micronutrient in lung development. A lower maternal haemoglobin concentration during pregnancy is associated with elevated IgE and increased risk of allergic sensitisation in the offspring [6]. Furthermore, maternal anaemia limits foetal intrauterine iron supply [1,7], which may have implications for foetal lung development. Post-partum, infant growth velocity is significantly correlated with iron deficiency anaemia in the first two years of life [8]. However, beyond these studies that associate maternal iron deficiency and increased risk of respiratory disease, no studies have demonstrated that low maternal systemic iron status during pregnancy alters lung development and function, whilst increasing the risk of asthma and disease severity, in offspring.

Given the high proportion of women affected by iron deficiency in pregnancy globally and the links with respiratory disease, it is critical to enhance our understanding of how lower maternal iron status during pregnancy affects respiratory disease in the offspring and determine whether these changes can be prevented and/or mitigated. To address this, we placed mice on a low iron diet (LID) prior to, and maintained throughout, pregnancy to induce iron deficiency [9], then studied house dust mite (HDM)-induced asthma in the offspring to investigate how lower iron status during pregnancy impacts lung function, structure and inflammatory responses.

## 2. Materials and Methods

### 2.1. Animal Ethics Statement

Animal procedures were performed in accordance with the Australian Code of Practice for the Care and Use of Animals for Scientific Purposes as issued by the National Health and Medical Research Council (NHMRC) Australia and approved by The University of Newcastle Animal Care and Ethics Committee (A-2019-941, approved 6 December 2019).

### 2.2. Mice

Wild-type BALB/c mice were sourced from the Central Animal House of The University of Newcastle (Callaghan, NSW, Australia). Animals were housed under specific pathogen free (SPF), PC2 conditions in individually ventilated cages (Bioresources facility, HMRI Building, New Lambton Heights, NSW, Australia). All mice were provided with food and water *ad libitum*.

### 2.3. Low Systemic Iron Diet during Pregnancy and Time Mating Protocol (F0 Generation)

**Figure 1:**
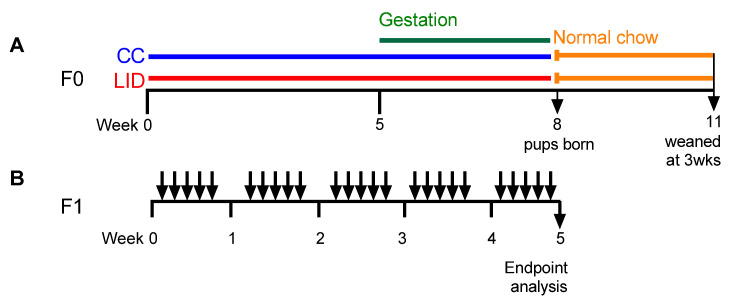
Protocols. (**A**) Six to eight-week-old, female wild type BALB/c mice (F0) received control chow (CC) or low iron diet (LID) from week 0. Timed mating occurred at week five and offspring weaned at three weeks of age. (**B**) Eight-week-old female offspring (F1) treated with house dust mite (HDM; 25 μg/50 μL Saline) or saline (Sal; 50 μL) five days/week for five weeks.

Six to eight-week-old female, wild-type BALB/c mice, received either a LID (~2.5 mg Fe/kg, SF01-017 diet based on AIN-93G, <5 mg Iron/Kg, Specialty Feeds, Glen Forrest, Australia) to induce systemic iron deficiency [9] or a control chow diet (CC, SF09-091, 75 mg Iron/Kg, Specialty Feeds, Glen Forrest, Australia) commencing 5 weeks (Figure 1A) prior to mating. Timed mating periods were conducted by firstly synchronising the induction of oestrous cycles at week five of the protocol. Males (8–10 weeks old) were housed individually for seven days before being removed to new, clean cages. Female mice were introduced to the dirty cages previously occupied by the males (two females per male cage) for three days before being removed and introduced to the new, clean cages occupied by the males (two females per male). Mating occurred over three days and presence of mucus plugs were checked daily. Females with plugs were transferred into individual housing in clean cages for the duration of pregnancy and the birth and weaning of offspring. The status of pregnant females was monitored by regular weighing, and births occurred during weeks eight of the protocol.

Post-partum, all mice were placed onto a normal mouse chow (meat free mouse and rat diet, 200 mg Iron/Kg Specialty Feeds, Glen Forrest, Australia) and offspring (F1) were weaned at three weeks of age.

### 2.4. Establishment of HDM-Induced Experimental Asthma (F1 Generation)

HDM-induced experimental asthma was induced in the F1 generation at eight weeks of age via intranasal administration of house dust mite (HDM) extract (25 µg, *Dermatophagoides pternyssinus* extract FD, Citeq biologics, Groningen, Netherlands) in medical grade sterile saline (50 µL), once daily, five times per week, for five weeks under isoflurane anaesthesia (Figure 1B). Due to a female-biased offspring sex ratio in the F1 generation, the experiments that examined the effects of maternal LID on asthma severity were conducted in female F1 offspring only.

### 2.5. Assessment of Airway Inflammation

Airway inflammation was measured in the bronchoalveolar lavage fluid (BALF) as previously described [9,10]. Briefly, the right multi-lobes of the lung were tied off, and the left lung was flushed with Hank’s Buffered Salt Solution (HBSS, 2 × 500 µL). Samples were centrifuged (5 min, 132× *g*, 4 °C), and the cell pellets were re-suspended in red cell lysis buffer (200 µL, 5 min on ice), spun down and cell pellets were re-suspended in HBSS (200 µL). The proportion of viable cells was assessed using the trypan blue exclusion method by counting using a haemocytometer. Cell suspensions (90 µL) were spun onto slides using cytocentrifugation (5 min, 300 rpm, room temperature). Differential leukocyte populations were determined under a light microscope at 400× magnification, following May Grunwald-Giemsa stain as per the manufacturer’s instruction (Sigma-Aldrich, Castle Hill, Australia).

### 2.6. Lung Function Analyses

Lung function tests were administered as previously described [9,10]. Briefly, mice were anaesthetised by intraperitoneal administration of ketamine (400 mg/kg; Parnell) and xylazine (10 mg/kg; Troy Laboratories, Glendenning, Australia) in saline (200 µL final volume) prior to tracheotomy and cannulation. Mice were ventilated at 450 breaths/min, and lung function parameters were assessed using the FlexiVent apparatus (FX1 System; SCIREQ^TM^, Montreal, Canada) with increasing doses of nebulised methacholine (0, 0.1, 1, 3 and 10 mg/mL) as previously described [9,10]. A minimum of three measurements per dose was performed for each parameter. Data were analysed using Flexiware software v7.6 (SCIREQ^TM^, Montreal, Canada), Microsoft Excel v10 (Microsoft, Redmond, WA, USA) and GraphPad v8 (Graphpad Software Inc., San Diego, CA, USA).

### 2.7. Histological Analyses

Following collection of BALF, the left lobes of the lungs were perfused with 0.9% saline and inflated with, and drop-fixed in, 10% neutral buffered formalin (up to 500 µL; Sigma-Aldrich, Castle Hill, Australia), paraffin embedded, sectioned and stained (HMRI histology services, New Lambton Heights, Australia).

Alveolar diameter was measured using Haematoxylin and Eosin-stained slides; The number of mucus secreting cells (MSCs)/μm of basement membrane (BM), using Alcian Blue Periodic Acid-Schiff (AB PAS) stain; Small airway collagen deposition, using Sirius Red/Fast Green stain; The number of eosinophils/100 μm of the basement membrane using Congo-Red (CR); as previously described [9,10,11]. Images were captured using a Zeiss AxioImager.M2 microscope (Zeiss Australia, North Ryde, NSW, Australia) and Zeiss ZEN software (v3.2, Zeiss Australia, North Ryde, Australia) and analysed using custom scripts in ImageJ v1.5 (NIH, Bethesda, MD, USA) as previously described [9,10,11].

### 2.8. Statistical Analyses

All data are presented as mean ± S.E.M. Comparisons between two groups were made using an unpaired students *t*-test. Comparisons between multiple groups were performed using a one-way analysis of variance (ANOVA) with a post-hoc Fisher’s least significant difference (LSD) test. Airway hyperresponsiveness (AHR) data were analysed using two-way repeated measures ANOVA with a post-hoc Tukey test. All statistical analyses were performed using GraphPad Prism software v8 (Graphpad Software Inc., San Diego, CA, USA).

## 3. Results

### 3.1. Low Iron Diet (LID) during Pregnancy Impairs Lung Function, Increases Airway Inflammation, and Increases Small Airway Collagen Deposition in Offspring

**Figure 2:**
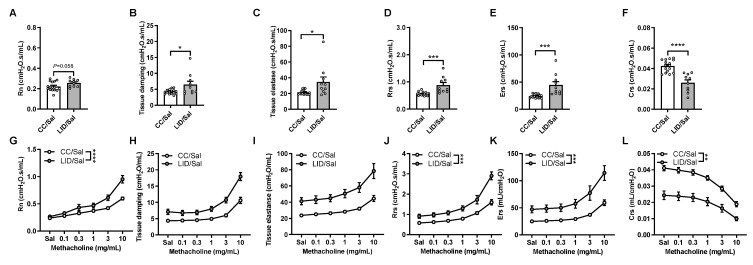
Low iron diet (LID) during pregnancy increases airway hyperresponsiveness (AHR), indicative of impaired lung function, in male and female offspring. Baseline lung function was assessed in terms of (**A**) central airway resistance (Rn), (**B**) tissue damping, (**C**) tissue elastance, (**D**) transpulmonary resistance (Rrs), (**E**) elastance (Ers), and (**F**) compliance (Crs). The response to methacholine provocation was assessed in terms of (**G**) Rn, (**H**) tissue damping, (**I**) tissue elastance, (**J**) Rrs, (**K**) Ers, (**L**) Crs. (**A**–**F**) Analysed by unpaired student *t*-test, (**G**–**L**) analysed by 2-way ANOVA and statistics at maximal dose from AHR curves presented. *n* = 10–16 mice per group. Data are presented as mean ± SEM (* *p* < 0.05; ** *p* < 0.01; *** *p* < 0.001; **** *p* < 0.0001).

To determine whether lower iron status during pregnancy alters lung function in the offspring, mice were fed an LID or CC [9], time mated at week five, the offspring weaned at three weeks of age, and treated with saline five days/week for five weeks (Figure 1A,B). Baseline lung function measurements and response to methacholine provocation were assessed. An LID during pregnancy increases baseline central airway resistance (Rn; *p* = 0.058; Figure 2A), tissue damping (Figure 2B), tissue elastance (Figure 2C), transpulmonary resistance (Rrs; Figure 2D) and transpulmonary elastance (Ers; Figure 2E) and decreases compliance (Crs; Figure 2F) in saline-treated offspring (LID/Sal), compared to saline-treated offspring from mothers on a CC (CC/Sal). We next measured the response to methacholine provocation to assess AHR in the offspring (Figure 2G–L). An LID during pregnancy increases AHR in terms of Rn, Rrs, Ers and decreases Crs in Saline-treated offspring (LID/Sal), compared to those from mothers on a CC (CC/Sal; Figure 2G,J–L). An LID during pregnancy had no effect on tissue damping or elastance (Figure 2H,I).

**Figure 3:**
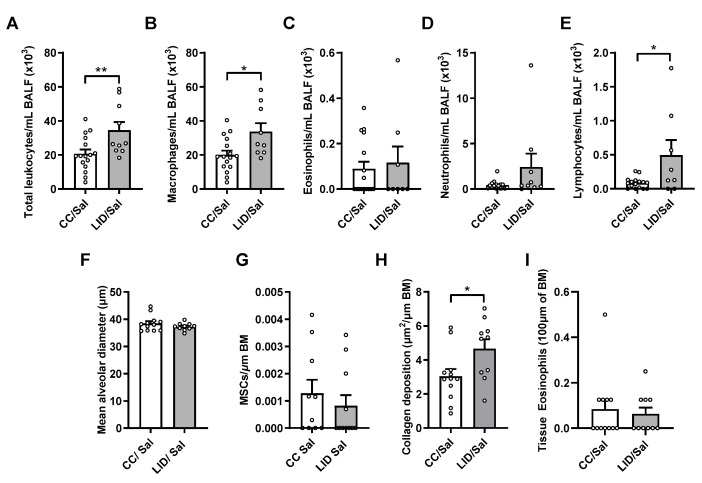
Low iron diet (LID) during pregnancy increases airway inflammation and collagen deposition in male and female offspring. (**A**) Total leukocytes, (**B**) macrophages, (**C**) eosinophils (**D**) neutrophils and (**E**) lymphocytes were enumerated in bronchoalveolar lavage fluid (BALF) at five weeks. Histopathology was assessed in terms of (**F**) mean alveolar diameter, (**G**) mucus secreting cells (MSCs) per μm of basement membrane (BM), (**H**) small airway collagen deposition and (**I**) airway-associated eosinophils. n = 10–16 mice per group. Analysed by unpaired student *t*-test. Data are presented as mean ± SEM (* *p* < 0.05; ** *p* < 0.01).

To determine whether an LID during pregnancy altered airway inflammation in male and female offspring, BALF was assessed for total leukocytes, macrophages, eosinophils, neutrophils and lymphocytes (Figure 3A–E). An LID during pregnancy significantly increases total leukocyte, macrophage, and lymphocyte numbers in the airways of saline-treated offspring (LID/Sal), but not neutrophils or eosinophils, compared to mothers on a CC (CC/Sal; Figure 3A–E). To assess lung structure in the offspring, alveolar diameter, mucus-secreting cells, collagen and airway-associated eosinophils were assessed. An LID during pregnancy does not affect alveolar diameter (Figure 3F) or mucus-secreting cells (Figure 3G) but increases small airway collagen deposition (Figure 3H). Furthermore, there are no changes in tissue eosinophil numbers around the small airways (Figure 3I). Together, these data show that LID during pregnancy impairs lung function, increases airway inflammation and increases collagen deposition around the small airways in the offspring.

### 3.2. Low Iron Diet (LID) during Pregnancy Impairs Lung Function, Increases Airway Inflammation, and Increases Small Airway Collagen Deposition in Female Offspring Treated with HDM

To assess whether an LID during pregnancy affects HDM-induced experimental asthma in female offspring (Figure 1A,B), lung function, airway inflammation and lung structure were assessed.

**Figure 4:**
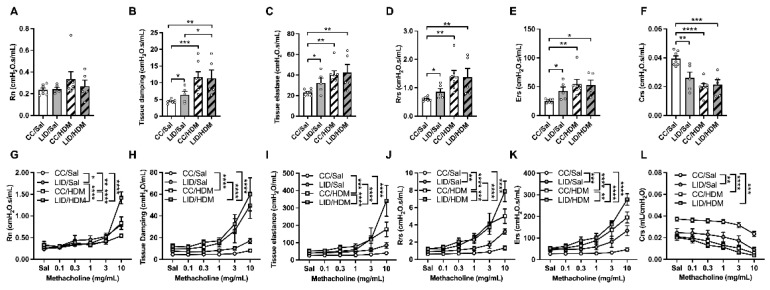
Low iron diet (LID) during pregnancy impairs lung function and increases AHR in female offspring in the absence and presence of HDM. Baseline lung function was assessed in terms of (**A**) central airway resistance (Rn), (**B**) tissue damping, (**C**) tissue elastance, (**D**) transpulmonary resistance (Rrs), (**E**) elastance (Ers), (**F**) compliance (Crs). Response to methacholine provocation was assessed in terms of (**G**) Rn, (**H**) tissue damping, (**I**) tissue elastance, (**J**) Rrs, (**K**) Ers, (**L**) Crs. (**A**–**F**) Analysed by one-way ANOVA with Fishers LSD and unpaired student *t*-test, (**G**–**L**) analysed by 2-way ANOVA and statistics at maximal dose from AHR curves presented. n = 5–7 mice per group. Data are presented as mean ± SEM (* *p* < 0.05; ** *p* < 0.01; *** *p* < 0.001; **** *p* < 0.0001).

HDM-treated female offspring from mothers on a CC (CC/HDM) have similar baseline lung function to saline-treated offspring from mothers on an LID (LID/Sal; Figure 4A–F). HDM-treated offspring from mothers on an LID (LID/HDM) have increased tissue damping (Figure 4B), but no other parameters are affected, compared to saline-treated offspring from mothers on an LID (LID/Sal). We next measured the response to methacholine provocation to assess AHR in the offspring (Figure 4G–L). An LID during pregnancy increases AHR in terms of Rn, Rrs, Ers and decreased Crs in saline-treated offspring (LID/Sal), compared to those from mothers on a CC (CC/Sal; Figure 4G,J–L). A LID during pregnancy has no effect on tissue damping or elastance (Figure 4H,I). HDM-treated offspring from mothers on a CC (CC/HDM) have increased AHR compared to saline-treated offspring from mothers on a CC (CC/Sal; Figure 4G–L). Interestingly, HDM-treated offspring from mothers on a LID (LID/HDM) exhibited further increases in AHR in terms of Rn, elastance, Rrs and Ers, but no change in tissue damping or Crs, compared to HDM-treated offspring from mothers on a CC (CC/HDM; Figure 4G–L). Together these data show that saline-treated female offspring from mothers on an LID have decreased baseline lung function and increased AHR compared to female offspring from mothers on a CC. Furthermore, HDM-treated female offspring from mothers on an LID have further impairment of baseline lung function and increased magnitude of AHR compared to HDM-treated female offspring from mothers on a CC (Figure 4).

**Figure 5:**
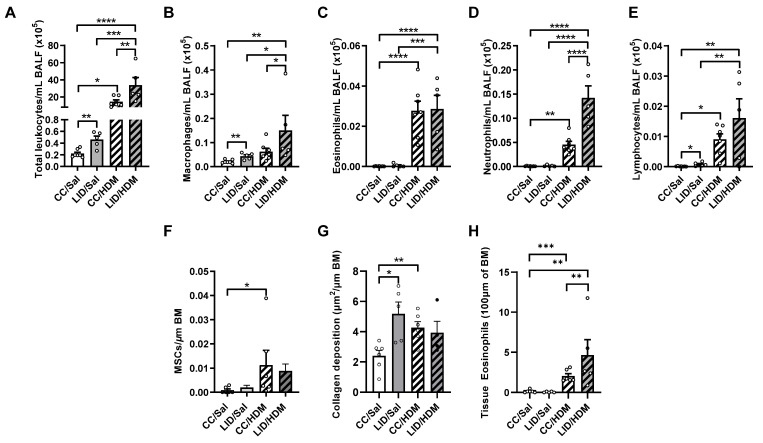
Low iron diet (LID) during pregnancy increases airway inflammation and collagen deposition in female offspring in the absence and presence of HDM. (**A**) Total leukocytes, (**B**) macrophages, (**C**) eosinophils (**D**) neutrophils and (**E**) lymphocytes were enumerated in bronchoalveolar lavage fluid (BALF) at five weeks. Histopathology was assessed in terms of (**F**) mucus secreting cells (MSCs) per μm of basement membrane (BM), (**G**) small airway collagen deposition and (**H**) airway-associated eosinophils. *n* = 5–7 mice per group. Data analysed by one-way ANOVA with Fishers LSD and unpaired student *t*-test Data are presented as mean ± SEM (* *p* < 0.05; ** *p* < 0.01; *** *p* < 0.001; **** *p* < 0.0001).

To determine whether an LID during pregnancy alters airway inflammation in control and HDM-treated female offspring, BALF was assessed for total leukocytes, macrophages, neutrophils, eosinophils, and lymphocytes (Figure 5A–E). An LID during pregnancy significantly increases total leukocyte, macrophage, and lymphocyte numbers in the airways of saline-treated offspring (LID/Sal), but not eosinophils or neutrophils, compared to saline-treated controls (CC/Sal; Figure 5A–E). HDM-treated female offspring from mothers on a CC (CC/HDM) have increased total leukocytes, eosinophils, neutrophils and lymphocytes, but not macrophages, compared to saline-treated female offspring from mothers on a CC (CC/Sal; Figure 5A–E). Interestingly, HDM-treated offspring from mothers on an LID (LID/HDM) had a further increase in total leukocytes, macrophages and neutrophils, and no change in eosinophils or lymphocytes, compared to HDM-treated offspring from mothers on a CC (CC/HDM; Figure 5A–E). Together, these data show that an LID during pregnancy increases airway inflammation in offspring, and that the inflammation associated with HDM-induced experimental asthma is further increased in offspring from mothers on an LID.

We next assessed whether an LID during pregnancy altered lung structure in control and HDM-treated female offspring. An LID during pregnancy had no effect on mucus-secreting cells (Figure 5F), however, mucus-secreting cells are increased in HDM-treated female offspring from mothers on a CC (CC/HDM) compared to saline-treated female offspring from mothers on a CC (CC/Sal; Figure 5F). An LID during pregnancy significantly increased small airway collagen deposition in saline-treated female offspring (LID/Sal; Figure 5G) to levels similar to those observed in HDM-treated female offspring from mothers on a CC (CC/HDM; Figure 5G). Furthermore, there are no changes in tissue eosinophil numbers around the small airways (Figure 5H). Together, these data show that an LID during pregnancy and HDM-treatment in offspring result in increased small airway collagen deposition.

## 4. Discussion

Iron dysregulation is strongly implicated in the development and/or exacerbation of many diseases. We and others [9,10,12,13,14,15,16,17] have previously shown that lung iron levels and their regulation play important roles in the pathogenesis and severity of lung infection and respiratory diseases such as asthma, chronic obstructive pulmonary disease, cystic fibrosis and idiopathic pulmonary fibrosis [9,10]. Furthermore, increasing clinical evidence suggests that maternal anaemia or an LID during pregnancy are linked with asthma and wheezing in offspring in later life [4]. However, no studies have demonstrated that low maternal iron status during pregnancy results in altered lung function, inflammatory response and structure, and whether this results in increased severity of asthma in offspring. Using our mouse models, we show for the first time that LID during pregnancy impairs baseline lung function, increases AHR, increases airway inflammation and promotes small airway collagen deposition in offspring. Furthermore, HDM-induced experimental asthma in the offspring of mothers on a LID during pregnancy was characterised by exaggerated features of disease, indicating that iron dysregulation during pregnancy imparts functional and structural changes in the lung of offspring in the presence or absence of experimental asthma.

It is known that maternal insults such as hypoxia that cause intrauterine growth restriction, lead to profound deleterious effects on lung and diaphragm function in offspring independent of sex and changes in lung structure [18,19]. Our findings now strongly suggest that LID in pregnancy is another significant maternal insult that can have profound deleterious effects on lung function in the offspring. A previously published study demonstrated that low foetal iron is linked to increased susceptibility to eosinophilia in infancy [20]. Here we show increased airway tissue eosinophils in asthmatic offspring from mothers on an LID diet. We also observed increases in small airway collagen deposition in offspring born to mothers on an LID, and it is known that collagen deposition is associated with AHR and airway remodelling in asthma [21,22,23]. Furthermore, we observed increases in macrophage and lymphocytes that may have also contributed to AHR in these offspring. Further studies that interrogate the mechanistic interplay between these features and how they contribute to increased AHR in offspring from mothers on an LID diet are warranted.

Our findings demonstrate that lung structure is altered by a low iron status *in utero*. This concept is supported by a study showing that desferrioxamine-mediated iron chelation in ex vivo lung buds from mouse embryos restricted the development of vascular networks and reduced epithelial branching [24]. Mothers in our study were on a maternal diet containing normal iron levels post-partum, demonstrating that the changes induced in the offspring in utero could not be reversed by a normal diet post-partum. This contrasts with a study that showed reversal of desferrioxamine-mediated iron chelation following iron exposure in mouse embryos [24]. Thus, in our model, post-partum maternal exposure to a diet containing normal iron levels did not correct the disease-causing effects of LID in utero on the offspring.

A longitudinal study in humans demonstrated that lower iron status in the umbilical cord was linked to increased risk of atopy in children in later life [25]. Our observations that HDM-treated offspring from mothers on an LID had increased disease features, including impaired lung function and airway inflammation are consistent with these clinical observations. Interestingly, our observation of increased AHR was not associated with further increases in mucus-secreting cells or collagen deposition around the small airways, but in this context the increase in AHR may be explained by the increases in total leukocytes and neutrophils observed in the airways of HDM-treated female offspring from mothers on a LID compared to mothers on a CC diet. Our findings demonstrate that an LID during pregnancy promotes a shift from classically eosinophil-dominated allergic airway inflammation to a mixed eosinophilic/neutrophilic inflammatory profile in HDM-induced allergic airway disease. We and others have shown that increased neutrophils in the airways in both clinical and experimental asthma is associated with more severe disease [26,27,28,29,30], indicating that responses to specific environmental exposures in adulthood play important roles in the development of severe disease. Here, we show that lower iron status in pregnancy results in increased neutrophilic inflammation during asthma in offspring, which is reminiscent of the increased neutrophilic responses that are observed in severe asthma [26,27,28]. Significantly, our study suggests that in utero responses to low iron status may be considered as another environmental exposure that has profound effects on the respiratory milieu of offspring, and that correcting low iron status in pregnancy may be beneficial for reducing the severity of asthma in the offspring. These findings suggest that studies that assess the links between low iron status in pregnancy and the prevalence and severity of asthma in the offspring may be warranted.

The limitations of our study include that LID during pregnancy was administered before and during pregnancy but not specifically during pregnancy and/or postpartum which may have additional impacts on disease in the offspring. Whilst our study examines the effects of maternal LID intake, further insights into the effects of lower iron status during pregnancy on disease development in offspring may be gained by testing the effects of diets with different concentrations of iron during pregnancy. It is known the iron deficiency can contribute to neurological changes through epigenetic mechanisms, however no studies to date have assessed the epigenetic alternations in the lungs of offspring born to mothers with LID during pregnancy. An examination of this relationship would be informative and warrants further investigation.

In addition, there is evidence to suggest that low iron can affect the composition of the gut microbiome and it is established that vertical transmission of the microbiome from mothers to offspring can occur [31,32,33]. These data present the possibility that maternal LID may alter the maternal gut microbiome and, through vertical transmission, may contribute to changes in offspring gut microbiome and lung changes.

Whilst such studies are beyond the scope of the current manuscript, future studies that investigate the role of epigenetics or microbiomes in driving the effects of low iron status in pregnancy on lung structure, function, and inflammatory responses in the lungs of offspring are warranted.

In our HDM-treated offspring from mothers on an LID, we used female mice; however, it would be informative to compare male vs. female in this context. Importantly, our observations in this study suggest that correcting iron deficiency during pregnancy with iron supplements and/or nutraceuticals may be beneficial for lung function and structure in the offspring, and this could be tested in intervention studies using our model. One such approach may be to boost iron intake through a measured nutraceutical approach involving dietary supplementation of iron, and/or point-of-use fortification, which has been proposed to be beneficial in chronic inflammatory lung diseases [34].

## 5. Conclusions

Our observations show that low iron status in pregnancy can have profound effects on lung function and increases the severity of disease features in offspring. Importantly, our study reconciles clinical observations that have linked lower iron levels in pregnancy and asthma risk in offspring by demonstrating a causal link. Our findings demonstrate how lower iron status in pregnancy affects respiratory disease and further support the importance of treating low iron during pregnancy with iron supplements. The mouse model of LID in pregnancy that we have developed recapitulates the effects of low iron in pregnancy on human respiratory disease, and it therefore provides not only an ideal platform for studying the disease-causing mechanisms of lower iron status in pregnancy but will also allow pre-clinical testing of the efficacy of iron-correcting interventions to be conducted.
